# A Survey of the Vitamin and Mineral Content in Milk from Yaks Raised at Different Altitudes

**DOI:** 10.1155/2021/1855149

**Published:** 2021-12-24

**Authors:** Lin Yang, Chen Yang, Fumin Chi, Xuedong Gu, Yahui Zhu

**Affiliations:** College of Food Science, Tibet Agriculture & Animal Husbandry University, Nyingchi 860000, China

## Abstract

In this study, the content of vitamins and of toxic and beneficial (macro- and micro-) minerals in milk from yaks raised at different altitudes (3,215, 4,340, and 5,410 m) was investigated. For comparison, the components in cow's milk were also measured. At higher altitudes, a significant (*P* < 0.05) increase in vitamin A and vitamin E was observed in the yak's milk, whereas the opposite was observed for vitamin B_1_ and vitamin B_2_. No significant statistical difference in vitamin C, Ca, P, Na, K, and Mg concentrations was observed in milk from yaks raised at different altitudes. The concentrations of Zn in milk from yaks raised at different altitudes showed no statistical difference, whereas the Mn and Fe concentrations in milk from yaks raised at 3,215 m were lower than those raised at higher altitudes. The concentrations of Pb and Cd in yak's milk did not exceed the maximum permissible concentrations (Codex Alimentarius Commission), whereas their concentrations were higher in milk from yaks raised at 3,215 m than at higher altitudes. These findings indicated that the contents of vitamins and minerals in yak milk varied in different altitudes.

## 1. Introduction

The yak (*Bos grunniens*) is a unique large ruminant livestock in the Qinghai-Tibet Plateau area, which has an altitude of 3,000 to 6,000 m and is located in western China. Yak's milk accounts for approximately 15% of China's milk production [1]. Yaks thrive in extremely harsh environments, under conditions including hypoxia, strong ultraviolet rays, minimal forage grass, and low temperatures [2]. Yak's milk is richer than cow's milk in the content of proteins, fat, and total solids; is easier to digest; and has lower allergenicity [3, 4]. It is virtually the sole source of milk in the Qinghai-Tibet Plateau area.

Because yak's milk is an important resource to the local population, a more comprehensive understanding of yak's milk is necessary. Many studies have been performed on the main constituents of yak's milk, including the chemical composition, the protein or fatty acid distribution, and the microbiological compositions [5–8]. However, little information is available on the minor constituents of yak's milk, mainly vitamins and minerals [9]. These two minor constituents play important roles in the physiological development of humans. Vitamins in milk mainly consist of V_A_, V_E_, and V_B_ (V_B1_ and V_B2_), which cannot be manufactured within the body and must be obtained from the diet. In the Qinghai-Tibet Plateau area, yak's milk is virtually the sole vitamin source for the local population. Although some research has examined V_A_ and V_c_ [10], information on the other important vitamins (V_B_ and V_E_) remains unavailable, and their differences in milk from yaks raised at different altitudes are unknown. Minerals in milk are divided into beneficial and toxic elements. The latter can cause metabolic disorders, even at low concentrations. The beneficial minerals have been examined in some studies [11, 12]; however, the toxic elements, particularly their differences depending on altitude, have not gained substantial research attention [10, 13, 14].

The objective of this study was to investigate the content of vitamins and of toxic and essential (macro- and micro-) metals in milk from yaks raised at different altitudes.

## 2. Materials and Methods

### 2.1. Sample Collection

Three sampling sites were selected in the Tibet Autonomous Region in the Qinghai-Tibet Plateau area: Zhongsha village in Gongbo'gyamda (29°14′47^″^N and 93°25′27^″^E, 3,215 m in altitude), Gongtang village in Damxung county (30°51′16^″^N and 91°05′29^″^E, 4,340 m in altitude), and Duoma village in Shuanghu county (31°10′08^″^N and 84°52′17^″^E, 5,410 m in altitude), with annual average temperatures of 3, 1.7, and -5.5°C, respectively.

At each sampling site, 70 lactating yaks were chosen according to their ages (5–7 years), group sizes (2–4), and milk lactation stages (50–100 days after the beginning of lactation), at which the yield of milk is highest. Each milk sample was placed into a 100 mL plastic tube. To inhibit microbial growth, 0.03% sodium azide was added to the samples. The samples were frozen and stored at -20°C before analysis.

For comparison, 15 cow's milk samples were collected from Xuanhan County in the city of Dazhou, Sichuan Province (31°75′09^″^N and 107°96′42^″^E, 1,082 m in altitude), which has an annual average temperature of 21.4°C. The cows were fed 75% forage and 25% crop residues year round.

### 2.2. Determination of General Chemical Composition

The fat content in milk was determined according to GB 5413.3-2010 [15]. Lactose content was determined according to GB 5009.8-2016 [16]. Total solids in milk were measured after treatment at 105°C for 5 h. The protein content was determined with the Kjeldahl method and calculated from the nitrogen content in milk with a 6.38 conversion factor.

### 2.3. Determination of Vitamin

The method of GB 5009.82-2016, with slight modification, was applied to determine the V_A_ and V_E_ content [17]. Briefly, 3 mL of the sample was added into a glass tube, and this was followed by addition of 6 mL ethanol and 3 mL of 8.9 mol L^−1^ NaOH solutions. After incubation in a water bath for 30 min, the samples were cooled in an ice bath, and 20 mL of 40% diethyl ether and 60% petroleum ether mixture (containing 0.01% butylated hydroxytoluene) was added. After samples were vortexed for 3 min, 20 mL of ice water was added into the tubes, which were then inverted 20 times. Then, the samples were centrifuged at 3000 g for 20 min (H1-16KR, Hunan Kecheng Instrument Equipment Co., Ltd., China). The fatty layer was dried under vacuum at 38°C with a rotary evaporator (RE-2000A, Shanghai Yarong Co., Ltd., China). Finally, the samples were dissolved in methanol. The content of V_A_ and V_E_ was determined with high-performance liquid chromatography (HPLC, Agilent 1100 Series), by using a C18 chromatographic column (Zorbax Eclipse XDB-C18, 150 × 4.6 mm, 5 *μ*m). The eluent was a methanol and water mixture (95 : 5). The flow rate was 1.0 mL min^−1^. The wavelengths for V_A_ and V_E_ were 323 and 292 nm, respectively.

The content of V_B1_ and V_B2_ was measured according to a previously reported method [18]. The chromatographic column and HPLC equipment were the same as those used for V_A_ and V_E_. The eluent consisted of 20% methanol, 0.3% triethylamine, and 2.6% glacial acetic acid. The wavelengths for V_B1_ and V_B2_ were 246 and 268 nm, respectively. The method of GB 5413.18–2010 was applied to determine the V_c_ content [19]. V_c_ can be oxidized into dehydroascorbic acid. Dehydroascorbic acid can react with o-phenylenediamine, thus forming fluorescent compounds, which were detected with a fluorospectrophotometer (RF-6000, Shimadzu, Kyoto, Japan).

### 2.4. Determination of Minerals

One gram of raw yak's milk was added into 20 mL nitric acid and was then digested for 15 min at 150°C in a microwave digestion system (MK-3, Xinyi Microwave Chemical Science and Technology Ltd., Shanghai, China). After being cooled to room temperature, the samples were diluted to 100 mL with deionized water.

An inductively coupled plasma optical emission spectrophotometer (iCAP 6000, Thermo Scientific, Waltham, USA) was used to measure the content of iron (Fe), calcium (Ca), copper (Cu), sodium (Na), potassium (K), manganese (Mn), magnesium (Mg), zinc (Zn), and phosphorus (P). An atomic absorption spectrometer (TAS-990, General Analysis Instruments Co. Ltd., Beijing, China) was used to determine the content of cadmium (Cd) and lead (Pb).

### 2.5. Statistical Analysis

The experiments were performed in at least triplicate. Analysis of variance was used to determine the significance of differences among treatments, with *P* < 0.05 indicating significance.

## 3. Results

### 3.1. General Composition

The content of total solids and fat in yak's milk increased with increasing altitude, whereas the protein and lactose content showed no statistical differences among altitudes. In yak's milk, all components were higher than those in cow's milk (Table 1). This result was consistent with those reported by Li et al. [20].

### 3.2. Vitamin Content

Vitamin A and vitamin E are found in different forms, but only retinol and *α*-tocopherol are considered important [21], because they exhibit higher biological activity than the other forms of vitamins A or vitamin E, respectively. The content of water-insoluble vitamins A (retinol) and vitamin E (*α*-tocopherol) in milk from yaks raised at different altitudes is shown in Table 2. At higher altitudes, a significant (*P* < 0.05) increase in vitamin A and vitamin E was observed in the yak's milk. Vitamin A cannot be synthesized by animals and must be obtained exogenously. *β*-Carotene, the main precursor of vitamin A, has been found to increase with altitude in pasture [22], thus potentially explaining why the vitamin A content is positively associated with altitude. Vitamin E decreases oxygen consumption and improves tissue oxygenation [23] and thus could raise the body's tolerance to the hypoxic environment in plateau areas. Higher vitamin E levels in milk from yaks raised at higher altitudes would be helpful to the local population consuming yak's milk, because higher altitudes are deficient in oxygen. In addition, the vitamin A and vitamin E content in yak's milk was higher than that in cow's milk.

The content of the water-soluble vitamins B_1_ (thiamin), B_2_ (riboflavin), and C (ascorbic acid) in milk from yaks raised at different altitudes is shown in Table 2. The content of vitamin B_1_ and vitamin B_2_ was significantly higher in milk from yaks raised at 3,215 m than at 4,340 or 5,410 m. These two vitamins play important roles in different metabolic processes, such as the development of tissues and the synthesis of corticoids in the human body [24]. The daily recommended intake for vitamin B_1_ and vitamin B_2_ in China is 2.0 mg and 2–4 mg, respectively, for adults. Therefore, a 1 L serving of yak's milk can meet the B_1_ and vitamin B_2_ needs for adults. The content of vitamin C in milk from yaks raised at lower altitudes was higher than those at higher altitudes.

### 3.3. Macro-, Micro-, and Toxic Mineral Elements

The concentrations of toxic mineral elements in yak and cow milk are shown in Table 3. The concentrations of Pb in yak's milk obtained at 3,215, 4,340, and 5,410 m were 10.7 ± 4.5, 2.3 ± 1.1, and 1.6 ± 0.8 *μ*g/L, respectively. The concentrations of Cd in yak's milk obtained at 3,215, 4,340, and 5,410 m were 36.2 ± 12.5, 14.4 ± 5.8, and 11.6 ± 4.3 *μ*g/L, respectively. In the Codex Alimentarius Commission, the maximum permissible concentration of Pb and Cd in all samples is 20 *μ*g/L and 500 *μ*g/L, respectively. Thus, the content of both Pb and Cd in milk from yaks raised at different altitudes did not exceed the maximum permissible limit. In addition, the Pb and Cd content in yak's milk was higher at lower altitudes. The presence of Pb and Cd in milk is closely associated with the environment, including atmospheric sedimentation, waste disposal, and motor exhaust [11, 12]. In the present study, the 3,215 m altitude regions are more industrialized and urbanized than the other regions at higher altitudes. Therefore, attention should be paid to possible toxicological risks in more industrialized and urbanized regions.

The concentrations of macroelements (Ca, P, Na, K, and Mg) in milk from yaks raised at different altitudes are shown in Table 3. There was no significant statistical difference in the Ca, P, Na, K, and Mg concentrations in milk obtained from yaks raised at different altitudes, although the concentrations were all much higher than those in cow's milk (except for Na). The microelement content (Cu, Fe, Mn, and Zn) in milk from yaks raised at different altitudes showed different trends. The concentration of Zn in milk from yaks raised at different altitudes showed no statistical difference, whereas the Mn and Fe concentrations in milk from yaks raised at 3,215 m were lower than those raised at higher altitudes. However, the concentration of Cu was markedly higher in milk from yaks raised at 3,215 m than at higher altitudes. The recommended dietary intake for Cu, Fe, Mn, and Zn is 700–900, 800–1,800, 1,900–2,300, and 8,000–11,000 *μ*g/kg body weight [25]. Our data indicated that yak's milk cannot meet the needs for these four microelements for adults.

## 4. Conclusions

At higher altitudes, significantly higher vitamin A and vitamin E content was observed in yak's milk, whereas the opposite was observed for vitamin B_1_ and vitamin B_2_. There was no significant statistical difference in the Ca, P, Na, K, and Mg concentrations in milk from yaks raised at different altitudes. The concentration of Zn in milk from yaks raised at different altitudes showed no statistical difference, whereas the Mn and Fe concentrations in milk from yaks raised at 3215 m were lower than those raised at higher altitudes. The Pb and Cd concentrations were markedly higher in milk from yaks raised at 3,215 m than at higher altitudes.

## Figures and Tables

**Table 1:** Composition (%) of milk from yaks raised at different altitudes.

Component	Altitude			Cow's milk
	3,215 m	4,340 m	5,410 m	
Total solids	16.0 ± 1.6^b^	17.4 ± 1.6^c^	18.8 ± 1.6^d^	13.1 ± 0.7^a^
Fat	5.3 ± 0.4^b^	6.7 ± 0.7^c^	7.0 ± 0.9^c^	4.1 ± 0.6^a^
Protein	5.0 ± 0.7^b^	5.2 ± 0.3^b^	5.8 ± 0.5^b^	3.4 ± 0.4^a^
Lactose	4.9 ± 0.5^b^	5.3 ± 0.3^b^	5.7 ± 0.4^b^	5.1 ± 0.3^a^

Values are mean ± standard deviation; means with different superscript letters within the same row are significantly different (*P* < 0.05).

**Table 2:** Content of vitamins in milk from yaks raised at different altitudes.

	Altitude			Cow's milk
	3,215 m	4,340 m	5,410 m	
Vitamin A (mg/L)	0.45 ± 0.19^b^	0.78 ± 0.27^c^	0.74 ± 0.24^c^	0.16 ± 0.06^a^
Vitamin E (mg/L)	1.82 ± 0.43^b^	1.97 ± 0.51^bc^	2.33 ± 0.49^c^	1.37 ± 0.31^a^
Vitamin B_1_ (mg/L)	4.15 ± 1.68^c^	2.14 ± 0.69^b^	2.81 ± 0.83^b^	0.85 ± 0.32^a^
Vitamin B_2_ (mg/L)	9.71 ± 2.83^c^	5.24 ± 1.49^b^	5.72 ± 1.64^b^	1.91 ± 0.56^a^
Vitamin C (mg/L)	32.80 ± 9.03^c^	37.32 ± 11.07^bc^	37.75 ± 8.03^b^	21.51 ± 6.14^a^

Values are mean ± standard deviation; means with different superscript letters within the same row are significantly different (*P* < 0.05).

**Table 3:** Content of minerals in milk from yaks raised at different altitudes.

	Altitude			Cow's milk
	3,215 m	4,340 m	5,410 m	
Ca (mg/L)	1417.8 ± 284.7^b^	1451.9 ± 156.3^b^	1564.5 ± 244.9^b^	1137.2 ± 162.5^a^
P (mg/L)	1034.0 ± 173.2^b^	1155.1 ± 193.1^b^	1186.9 ± 211.6^b^	928.4 ± 87.1^a^
Na (mg/L)	353.8 ± 62.4^a^	374.8 ± 78.3^a^	342.9 ± 69.0^a^	379.1 ± 48.7^a^
K (mg/L)	1577.1 ± 375.7^a^	1597.9 ± 469.9^a^	1537.9 ± 445.1^a^	1427.9 ± 233.2^a^
Mg (mg/L)	175.8 ± 38.2^b^	159.1 ± 33.0^b^	148.1 ± 35.1^b^	109.6 ± 28.2^a^
Cu (*μ*g/L)	159.1 ± 38.1^a^	92.7 ± 23.8^b^	101.7 ± 25.1^b^	173.8 ± 42.5^a^
Fe (*μ*g/L)	705.0 ± 96.8^b^	905.7 ± 142.7^c^	986.1 ± 112.7^c^	462.3 ± 58.1^a^
Mn (*μ*g/L)	57.5 ± 12.8^b^	37.8 ± 9.7^a^	32.8 ± 11.3^a^	29.5 ± 9.9^a^
Zn (mg/L)	7.3 ± 2.3^b^	8.9 ± 1.8^b^	8.4 ± 1.7^b^	2.8 ± 1.4^a^
Pb (*μ*g/L)	9.2 ± 4.5^b^	2.3 ± 1.1^a^	1.6 ± 0.8^a^	16.3 ± 6.7^bc^
Cd (*μ*g/L)	36.2 ± 12.5^b^	14.4 ± 5.8^a^	11.6 ± 4.3^a^	48.2 ± 14.7^b^

Values within rows with different superscript letters are significantly different from each other (*P* < 0.05).

## Data Availability

The data used to support the findings of this study are available within the manuscript.
